# Systematic review: the bioavailability of orally administered antibiotics during the initial phase of a systemic infection in non-ICU patients

**DOI:** 10.1186/s12879-021-05919-w

**Published:** 2021-03-20

**Authors:** Annemieke K. van den Broek, Jan M. Prins, Caroline E. Visser, Reinier M. van Hest

**Affiliations:** 1 Department of Internal Medicine, Division of Infectious Diseases, Amsterdam UMC, University of Amsterdam, Meibergdreef 9, 1105 AZ Amsterdam, The Netherlands; 2 Department of Medical Microbiology, Amsterdam UMC, University of Amsterdam, Meibergdreef 9, 1105 AZ Amsterdam, The Netherlands; 3 Department of Hospital Pharmacy, Division of Clinical Pharmacology, Amsterdam UMC, University of Amsterdam, Meibergdreef 9, 1105 AZ Amsterdam, The Netherlands

**Keywords:** Pharmacokinetics, Oral antibiotics, Bioavailability, Acute infection, Non-ICU patients

## Abstract

**Background:**

The systemic response to an infection might influence the pharmacokinetics of antibiotics. To evaluate the desired possibility of an earlier (< 24 h) IV-to-oral switch therapy in febrile non-ICU, hospitalized patients, a systematic review was performed to assess the effect of the initial phase of a systemic infection on the bioavailability of orally administered antibiotics in such patients.

**Methods:**

An electronic search was conducted in MEDLINE and Embase up to July 2020. Studies were selected when outcome data were collected during the initial stage of a febrile disease. Outcome data were (maximum) serum concentrations, time of achieving maximum serum concentration, and the area-under-the-plasma-concentration-time curve or bioavailability of orally administered antibiotics. Risk of bias was assessed.

**Results:**

We identified 9 studies on 6 antibiotics. Ciprofloxacin was the most frequently studied drug. Outcomes of the studies were heterogeneous and generally had a high risk of bias. Three small studies, two on ciprofloxacin and one on clarithromycin, compared the pharmacokinetics of febrile patients with those of clinically recovered patients and suggested that bioavailability was not altered in these patients. Other studies either compared the pharmacokinetics in febrile patients with reported pharmacokinetic values from earlier studies in healthy volunteers (*n* = 2), or provided no comparison at all and were non-conclusive (*n* = 4).

**Conclusion:**

There is a clear knowledge gap regarding the bioavailability of orally administered antibiotics in non-ICU patients during the initial phase of a systemic infection. Well-designed studies on this topic are necessary to elucidate whether patients can benefit from the advantages of an earlier IV-to-oral switch.

**Supplementary Information:**

The online version contains supplementary material available at 10.1186/s12879-021-05919-w.

## Background

Patients hospitalized with serious infectious diseases are in general initially treated with parenteral antimicrobial therapy. Guidelines recommend to switch to oral therapy only when the patient has been treated intravenously (IV) for at least 48–72 h and in case the clinical condition has improved and the fever has abated [1]. The question is whether or not patients can be switched to oral antibiotics earlier than 48 h, which recently has become subject of debate [2, 3]. Switching to oral therapy has been shown to lower the length of hospital stay, the risk of new infections and healthcare costs, without compromising clinical outcome [4]. If there is a possibility to shorten the current recommended duration of IV therapy, these benefits are likely to be achieved earlier.

The main reasons why IV therapy is favoured in the beginning of the treatment of seriously ill infectious patients are the short time of achieving maximum serum concentrations (Tmax) and the 100% bioavailability [5, 6]. Orally administered antibiotics must undergo absorption from the gut and first pass metabolism before entering the systemic circulation, often causing a bioavailability of less than 100%, resulting in delayed and lower maximum concentrations in blood and at the site of infection compared to IV administration. From a theoretical point of view, in case the gastrointestinal tract of the patient is intact and the bioavailability of an oral antibiotic agent is adequate, it should be possible to reach sufficient antibiotic exposure with orally administered antibiotics. However, the systemic response to an infection may alter the pharmacokinetics of antibiotics [7–10] and thus the bioavailability of oral antibiotics.

Acute infection-induced pathophysiological changes such as organ dysfunction and increased capillary permeability are known to lead to alterations in antibiotic volume of distribution and clearance [7–10]. In critically ill infectious patients, both toxic antibiotic serum concentrations due to renal hypoperfusion and acute kidney injury, and subtherapeutic antibiotic serum concentrations due to increased volumes of distribution and renal hyperperfusion, i.e. Augmented Renal Clearance (ARC), have been described [7–10]. Although data is limited, an effect on absorption and first pass effect cannot be ruled out in advance, as possible perfusion or other yet unknown alterations to the gastrointestinal tract may be present during the acute phase of infection [11]. The latter two pharmacokinetic parameters are particularly of relevance, since these determine the bioavailability of oral agents. The effect of infection on bioavailability may not necessarily be negative. Infection is associated with downregulation of the cytochrome P450 (CYP) enzymes, expressed by the liver and intestines, and responsible for drug metabolism and the first pass effect. This could lead to higher maximum concentrations in blood and the site of infection of CYP-dependent antimicrobials [12].

To date, the pharmacokinetics of antibiotics have mainly been tested in healthy volunteers or critically ill patients. Reports on the pharmacokinetics in the early infectious phase of non-ICU hospitalized patients are limited. In particular, data on oral bioavailability of antibiotics in this phase of disease are scarce and contradictory [11, 13, 14]. Consequently, we do not know whether adequate antibiotic levels can be reached in the systemic circulation when antibiotics are administered orally during the initial stage of an infectious illness. Hence, the recommended 48 h IV antibiotic treatment.

The aim of this study was to conduct a systematic review to assess the bioavailability of orally administered antibiotics during the initial phase of a systemic infection in non-ICU patients. The results may provide information whether starting with oral therapy or an earlier (< 24 h) IV-to-Oral switch might be possible and may guide future treatment policy and clinical research.

## Methods

### Protocol

This study was performed and reported according to the PRISMA (Preferred Reporting items for Systematic reviews and Meta-analysis) statement (Supplemental Table 1) [15].

### Eligibility criteria

Studies reporting data on the pharmacokinetics of oral antibiotics in the early phase of infection were searched, preferably, but not necessarily, in comparison with the convalescence phase of infection. Studies were eligible if they included patients aged 16 years or above and febrile or acutely ill due to an infectious disease, which had to be clearly documented or illustrated with elevated infectious laboratory parameters, i.e. CRP, leucocytosis, SIRS or qSOFA criteria [16]. We chose a subset of antibiotics which are widely used and known to have a moderate to good bioavailability, namely amoxicillin, flucloxacillin, ampicillin, clindamycin, macrolides, fluoroquinolones, metronidazole and trimethoprim-sulfamethoxazole. The pharmacokinetic outcome parameters of interest were those related to oral bioavailability: the (maximum) serum concentrations (Cmax), time of achieving maximum serum concentrations (Tmax), the area-under-the-plasma-concentration-time curve (AUC) or bioavailability itself (F). Blood samples for these outcome parameters had to be taken at the first day of antibiotic therapy, when patients were in the initial phase of their infectious disease. Intravenously pre-treated patients were excluded. Studies had to be reported in English or Dutch. We allowed all clinical study types, as long as they presented sufficient information to retrieve the patient inclusion criteria and the predefined outcome parameters. We excluded studies investigating healthy volunteers; patients admitted to the Intensive Care Unit; patients with impaired renal or hepatic function, because the impairment itself can already influence the predefined outcome parameters; and febrile neutropenic patients, because mucosal injury might make its findings not be generalizable to the general population.

### Search strategy

Together with an experienced clinical librarian, we conducted a systematic literature search in OVID MEDLINE and EMBASE for all relevant studies up to July 2020, based on the predefined objectives and eligibility criteria. In addition, we searched the reference lists of retrieved reviews. The primary records obtained were imported and de-duplicated in EndNote (complete search strategies can be found in supplemental Table 2 and 3). One reviewer (A.v.d.B.) screened all the titles and abstracts, to identify studies that potentially met the eligibility criteria. 10% was randomly assigned to and independently screened by the other reviewers (R.M.v.H, C.E.V.,J.M.P) to ensure reliability and completeness. Differences in decision were resolved by consensus. We allowed a 2.5% margin of difference between the reviewers. If after discussion the difference remained more than 2.5%, all articles had to be screened by the other reviewers. Next, the full text articles of the potentially relevant studies were retrieved and assessed for eligibility by all reviewers. Any disagreement on inclusion of studies was discussed by all reviewers and resolved by consensus. Finally, the reference lists of the eligible articles were screened by A.v.d.B for additional suitable studies.

### Data extraction and quality assessment

A standard form was used to extract and summarize the predefined outcome data and data necessary for the quality/risk of bias assessment of the included studies. Study design, patient characteristics, predefined pharmacokinetic outcome parameters and conclusions were extracted by one reviewer (A.v.d.B.) and fully checked for accuracy by another reviewer (R.M.v.H). Discrepancies were resolved by discussion, together with the other reviewers if necessary. Next, the risk of bias of the included studies was assessed independently by three reviewers (A.v.d.B., R.M.v.H., J.M.P.), using an adjusted form of the Newcastle-Ottawa Quality Assessment Scale (NOS) for non-randomized studies, and the Cochrane risk of bias tool for Randomized Controlled Trials [17, 18]. Studies could score points (or stars) on three dimensions: Selection (max. 5 stars), Comparability (max. 2 stars) and Outcome (max. 3 stars). The more stars, the lower the risk of bias. We adapted sub questions of these domains to enable more appropriate quality evaluation for descriptive studies reporting pharmacokinetic parameters. The maximum NOS score was 10 and we considered the risk of bias high when the score was 5 or lower and low when above the median score of 5.

## Results

### Search results

**Fig. 1:**
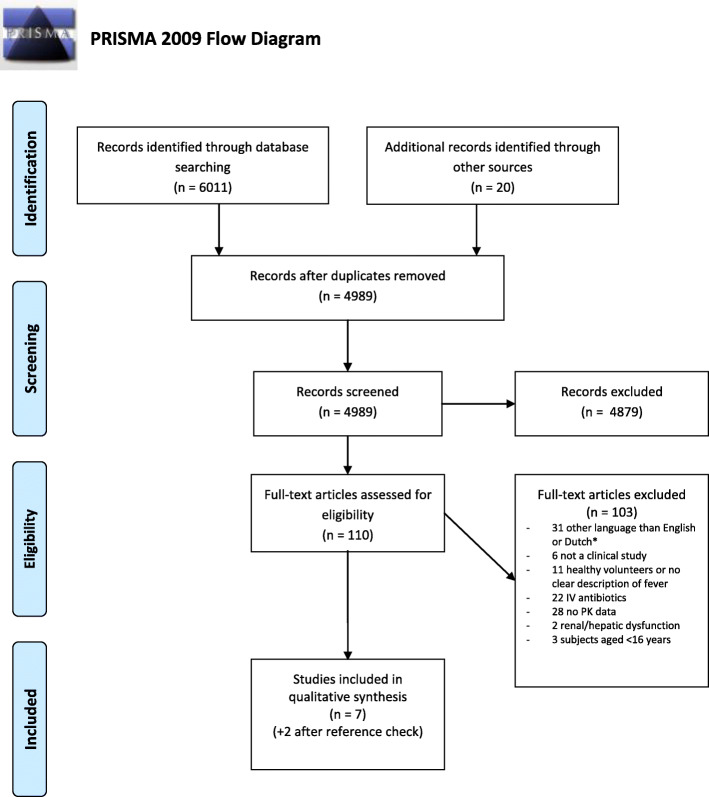
PRISMA 2009 flow diagram [30]

Our literature search yielded 6011 potentially relevant studies. After removing the duplicates 4989 papers remained. Based on the eligibility criteria, 4879 studies were excluded in the initial screening phase based on title and abstract, leaving 110 records for full text screening, including two records which were added after reference screening of two reviews addressing the pharmacokinetics of ciprofloxacin and intracellular pharmacokinetics of antibiotics [19, 20]. Of these, 103 were excluded, and 7 included after full-text screening. In addition, we identified 2 papers by reviewing the reference lists of the included studies, resulting in 9 papers for qualitative analysis [21–29]. (Fig. 1).

### Study characteristics

The study characteristics of the 9 included studies are presented in Table 1. The studies were non-randomized, observational studies [21–29] All studies were performed in hospitalized patients. 6/9 studies reported fever or other signs of acute phase of febrile illness [21, 25–29]. In 3/9 studies, which were the studies that were added after reference screening of the retrieved reviews and the reference lists of the included studies, the febrile state of patients was unclear, which may explain why these records were not captured in our initial search. However, the patients were hospitalized for acute purulent respiratory exacerbations and were in their initial phase of illness, making it highly likely that these patients were febrile or acutely ill as well. Therefore, we chose to include these studies [22–24]. Four studies originated from The Netherlands [21–24], two from the USA [27, 29], one from Canada [26], one from Egypt [25] and one from Guatemala [28]. The pharmacokinetics (PK) of amoxicillin was investigated by two studies [22, 25], the PK of azithromycin [21], ampicillin [22], clarithromycin [26] and enoxacin [24] by one study each and the PK of ciprofloxacin by four studies [23, 27–29].

**Table 1:** Characteristics of included studies

Study	Setting	Disease Phase of infection	Study design	Study drug Dosage regimen	Sample size Age (y)		Predefined outcomes						Conclusion	Risk of bias score
							Serum Concentrations or Cmax ( mg/l) Tmax (h) AUC (mg/l*h) Bioavailability (F in %)							
Farid et al, 1975 [25]	Hospitalized patients (Egypt)	Salmonella bacteraemia associated with Schistosomiasis Acute enteric fever/febrile	Cross-sectional PK after first dose on the first and second day of therapy	**amoxicillin** PO: 250mg qd	*N*=5 Age: 20-29 (N total:12, 7 with age<18 y)		**Day 1**			**Day 2**			Cserum day 2 adequate for the treatment of the Salmonellae isolated from the urine and blood.	4/10
							2h	3h	4h	2h	3h	4h		
						Cserum								
						Pat 7	0.12	4.1	3.1	3.5	-	2.4		
						Pat 8	2.0	1.1	-	3.3	-	0.7		
						Pat 9	7.1	-	2.1	8.5	-	1.15		
						Pat 10	3.1	3.25	1.0	0.63	-	2.0		
						Pat 11	3.5	3.95	3.65	1.57	-	3.9		
Davies et al, 1979 [22]	Hospitalized patients (The Netherlands)	Acute exacerbations of chronic bronchitis Initial phase	Cross-sectional PK after first dose of different antibiotics	**1. amoxicillin** PO: 750mg **2. ampicillin** PO: 1000mg	1. *N*=23 2. *N*=17 Age: -		**Amoxicillin**			**Ampicillin**			Amoxicillin measured serum concentrations generally satisfactory to treat H. influenza and S. pneumoniae Ampicillin does not yield satisfactory concentrations in serum and sputum.	1/10
						Cmax	11 (6-15 visual inspection)			8.3 (4-13 visual inspection)				
						Tmax	1.5			2				
						AUC	30.19			26.34				
Bohte et al, 1995 [21]	Hospitalized patients (The Netherlands)	CAP Initial phase	Cross-sectional PK around first and second dose	**azithromycin** PO: 500 mg bd on day 1, thereafter od during 4 days	*N*=8 Age: 32-75		**3h**	**12h**		**15h**			Low Cserum during the first 12h of treatment as compared to healthy volunteers.	3/10
						Cserum	0.06-0.25	0.03-0.12		0.28-0.55				
Offman et al, 2000 [26]	Hospitalized patients (Canada)	CAP Acutely ill	Longitudinal cohort PK after first dose acutely ill vs. convalescent phase	**clarithromycin** PO: 500mg single dose	*N*= 12 Age: 77±2		**Acutely ill C**^**a**^			**Convalescent C**			No impaired oral absorption in acutely ill patients with CAP. During acute phase of significantly decreased Cmax and AUC of 14-hydroxy clarithromycin.	9/10
						Cmax	4.32 ± 0.63			3.57 ± 0.46				
						Tmax	3.50 ± 0.5			2.83 ± 0.59				
						AUC	47.37 ± 8.51			36.22 ± 6.09*				
							**Acutely ill 14-OH**^**b**^			**Convalescent 14-OH**				
						Cmax	0.42 ± 0.08			0.76 ± 0.23*				
						Tmax	4.83 ± 1.29			3.08 ± 0.51				
						AUC	5.84 ± 1.08			8.84 ± 1.92*				
Patel et al, 1995 [27]	Hospitalized patients (USA)	Acute infectious illnesses Acute febrile phase (oral T>38.9;rectal T>38.3)	Longitudinal cohort PK after first dose acutely ill vs. convalescent phase	**ciprofloxacin** PO: 500mg single dose	*N*=12 Age: 36 (20-62)		**Acutely ill**			**Convalescent**			No significant PK differences between acutely ill and convalescent phase.	8/10
						Cmax	2.45 (±0.77)			2.31 (±1.26)				
						Tmax	1.48 (±0.75)			2.48 (±1.46)				
						AUC	10.91 (± 3.64)			11.05 (± 4.41)				
Ramirez et al, 1985 [28]	Hospitalized patients (Guatemala)	Selected susceptible gram-negative or gram-positive infections Initial febrile phase, or febrile phase after inadequate treatment	Cross-sectional PK at first, fourth and last day of therapy	**ciprofloxacin** PO: 500mg bd	*N*= 71 (N total = 100) Age: 38.1 (18-84)		**Day 1:**	**Day 4:**		**Last day:**			Drug levels in blood lower than previously reported	4/10
						Cpeak	0.77 ± 0.43	0.79 ± 0.49		0.80 ± 0.41				
						Cthrough	0.29 ± 0.24	0.34 ± 0.32		0.29 ± 0.24				
Guay et al, 1987 [29]	Hospitalized patients (USA)	Lower respiratory tract infections Acutely ill	Longitudinal cohort PK after first dose acutely ill vs. convalescent phase	**ciprofloxacin** PO: 750mg bd	Febrile *N*= 7 Afebrile *N*=4 Age: 77.7 (71-89) (N total = 13, but 6 with renal/hepatic impairment)		**Acutely ill**			**Convalescent**			No significant PK differences between acute illness and convalescent phase.	6/10
							PK *n*= 4:^c^							
						Cmax	6.11 ± 2.67			9.9 ± 3.65				
						Tmax	1.6 ± 0.45			1.3 ± 0.6 h				
							PK *n*=7:							
						Cmax:	6.83 ± 3.39							
						Tmax:	1.8 ± 0.7							
Davies et al, 1986 [23]	Hospitalized patients (The Netherlands)	Acute purulent exacerbations of chronic bronchitis Initial phase	Cross-sectional PK after first dose of 3 different doses	**ciprofloxacin** PO: Group 1: 500mg bd Group 2A: 750mg bd (badge 1) Group 2B: 750mg bd (badge 2) Group 3: 1000mg bd	*N*=80 (8) Group 1: 20 Age: 66.2 Group 2A: 20 Age: 66.8 Group 2B: 20 Age: 60.3 Group 3: 20 Age: 65.9		**Group 1**		**Group 2A**	**Group 2B**		**Group 3**	Measured serum concentrations were generally satisfactory to treat H. influenza or B. catarrhalis.	3/10
						Cmax	3.36 (range 1-6)		2.3 (range 1.4-3.4)	3.13 (range 1.3-5)		3.76 (range 2.5-6)		
						Tmax	2.4		1.68	2.25		1.95		
						AUC	12.9 (range 6-20.7)		11.1 (range 7-15.6)	14.7 (range 6.8-25.6)		17.9 (range 9.9-25.8)		
Davies et al, 1984 [24]	Hospitalized patients (The Netherlands)	Acute exacerbations of chronic bronchitis Initial phase	Cross-sectional PK after first dose	**enoxacin** PO: 600mg bd	*N*= 15 Age: 66.4 (50-81)	Cmax Tmax AUC_0-7_ AUC_0-12_	3.7 (± 1.2 visual inspection) 2.3 17.03 25.02						Good GI-absorption. However, unclear comparator. The interpatient serum concentrations did not differ widely.	**1/10**

*Statistically significant differences: AUC^a, b^*p* < 0.10 for normally distributed values; Cmax^b^*p* < 0.05 for non-normally distributed values

^a^*C* Clarithromycin, ^b^14-hydroxy-clarithromycin

^c^Mean PK of the 4 patients who were also evaluated during the convalescent phase

### Quality assessment

The risk of bias results are listed in Table 2. Six out of nine studies had a low score and therefore a high risk of bias, mainly due to the sample selection, in which a sample size calculation was missing, and the outcome measurement, in which a clear description of the laboratory procedures for the measurement of drug concentrations was missing [21–25, 28].

**Table 2:** Quality and Risk of Bias Assessment

	OBSERVATIONAL STUDIES						
Study	Sample selection criteria			Comparability	Outcome & evaluation		Summary score/10
	*Representativeness sample:* 2 points if the sample is a truly representative of the average in the target population; 1 point if the sample is somewhat representative of target population; no points if unclear or no description	*Sample size:* 1 point is sample size is justified by using power analysis; no points if not justified	*Ascertainment of disease state or diagnosis:* 2 points if validated or accepted tool was used; 1 point if non-validated or non-accepted, but well described; no points if unclear or no description	*Comparability:* 1 point if group design and groups are comparable; no points when groups are not comparable, 1 point when no comparative design was used; 1 point if the study controls for possible confounders	*Assessment outcomes:* 1 point if appropriate blood collection/drug concentration measurement and laboratory procedures used; No points if unclear or no description of procedure	*Statistical test (analysis of outcomes)*: 2 points if population pharmacokinetic modelling with co-variate analysis or conventional 2-stage method with co-variate analysis or non-compartmental analysis with rich sampling (≥3/dosing interval); 1 point if outcome variables summarized while expressing variability; no points if the statistical test is unclear, incomplete or not described	
Bohte, 1995 [21]	★	–	–	★	–	★	3/10: low
Davies, 1986 [23]	–	–	–	★	–	★★	3/10: low
Davies, 1984 [24]	–	–	–	★	–	–	1/10: low
Davies, 1979 [22]	–	–	–	★	–	–	1/10: low
Farid, 1975 [25]	★	–	★	★	–	★	4/10: low
Guay, 1987 [29]	★	–	–	★★	★	★★	6/10: high
Offman, 2000 [26]	★	★	★★	★★	★	★★	9/10: high
Patel, 1995 [27]	★	★	★	★★	★	★★	8/10: high
Ramirez, 1985 [28]	★	–	★★	★	–	–	4/10: low

### Pharmacokinetic parameters in infectious patients during their initial state of disease

#### Amoxicillin and ampicillin

Two studies reported the PK of oral amoxicillin [22, 25], of which one study reported the PK of oral ampicillin as well [22]. The first study reported the mean Cmax, Tmax and AUC of amoxicillin (*n* = 23) and ampicillin (*n* = 17) measured in serum and sputum on the first day of therapy in patients diagnosed with acute respiratory exacerbations. The serum concentrations were plotted in a figure, from which we estimated the range of the Cmax through visual inspection [22]. The other study concerned patients with Salmonella typhi or paratyphi A bacteriuria and recurrent bacteraemia associated with schistosomiasis. All patients had acute enteric fever or were febrile. Only 5/12 patients were aged > 16 years, but since amoxicillin concentrations were reported individually, these five patients could be included. The authors reported the measured serum concentrations during the first 4 h after dose administration on day 1 and day 2 [25]. Both reports conclude that the measured serum and sputum concentrations of amoxicillin should be generally satisfactory for treatment, based on the minimum inhibitory concentration (MIC) values of the isolated pathogens, whereas ampicillin did not yield satisfactory concentrations in serum and sputum. However, none of the studies drew a clear conclusion on the bioavailability of oral amoxicillin or ampicillin during the febrile period of illness compared to the convalescence phase.

#### Azithromycin

One study reported the PK of azithromycin [21]. Although the report does not present the study population characteristics, the patients were derived from another trial, in which the state of disease was clearly described [31]. The total serum concentrations were measured at two time points within the first dosing interval in eight subjects. In five of them, one concentration was also measured after the second dose. The wide range of observed concentrations measured 3 h after the first dose (0.06–0.25 mg/l) indicate high inter-patient variability. The authors’ conclusion, that the initial phase of infection resulted in low serum levels during the first 12 h of illness, was based on a comparison with previously reported Cmax levels in healthy volunteers, ranging from 0.4–0.45 mg/l [21]. However, this conclusion is based on only two PK measurements per dosing interval, which increases the risk that the true Cmax and Tmax could not be accurately estimated. Also, the number of subjects (*n* = 8) seems not sufficient to draw a sound conclusion on the bioavailability of azithromycin during the initial stage of an infectious disease.

#### Clarithromycin

One study reported the PK of a single dose of clarithromycin in 12 patients diagnosed with community- acquired pneumonia, when they were acutely ill and after convalescence [26]. The AUC of clarithromycin was higher during the febrile phase compared to the afebrile phase, 47.37 μg/h/ml vs 36.22 μg/h/ml respectively (*p* = 0.075), which the authors considered significant based on a significance level set at 10%. No significant differences were found in Cmax and Tmax between the two phases. Therefore, the febrile phase of illness did not seem to impair the extent of oral absorption of clarithromycin. The concentrations of its metabolite were significantly decreased during this phase. An explanation may be that the infection altered the hepatic blood flow, impairing the first pass effect, which is reported to be strong for clarithromycin [32]. It would be interesting to know whether the patients were hypotensive or had a significantly different blood pressure between the two measurement days to strengthen this hypothesis.

#### Ciprofloxacin and enoxacin

Four studies investigated the PK of ciprofloxacin [23, 27–29]. One study reported the PK of enoxacin [24]. Patel and colleagues measured the Cmax, Tmax and AUC of a single oral dose of ciprofloxacin in patients diagnosed with acute infectious illnesses of any kind when they were acutely ill, compared to when they were afebrile. In this study, with a low risk of bias, no significant PK differences were seen between the two phases [27]. The study of Guay and colleagues also analysed the Cmax, Tmax and AUC of ciprofloxacin in the febrile phase compared with the afebrile phase, but mainly in patients diagnosed with lower respiratory tract infections. Again, no significant PK differences were seen between the two phases and also this study had a low risk of bias. However, 6/13 patients had impaired renal/hepatic function (i.e. cirrhosis and chronic liver disease). Because this study presented individual data of the subjects, patients with impaired renal/hepatic function were excluded and the remaining data of the eligible patients were summarized as described in the methods section of that study (Table 1). This left only 7 patients in the febrile phase, of which 4 patients were also studied in the afebrile phase, strongly limiting the power of the study [29]. The ciprofloxacin study by Ramirez measured the peak and trough serum concentrations during the initial disease phase (day 1), the fourth and last day of therapy [28]. On all three measurements days, the drug levels in these patients were lower than previously reported, yet there was resolution of the infectious process in 88 of 100 patients [33]. In addition, the mean serum levels did not differ between measurement days, so the infectious state of the patient did not seem to have an effect on the measured ciprofloxacin concentrations.The ciprofloxacin and enoxacin studies by Davies reported the Cmax, Tmax and AUC measured in serum and sputum concentration on the first day of therapy in patients diagnosed with acute respiratory exacerbations [23, 24]. In the ciprofloxacin study, the PK of different doses were studied (*n* = 20 per dosing group). In the enoxacin study (*n* = 15), the serum concentrations were plotted in a figure, from which we estimated the range of the Cmax through visual inspection. The authors concluded that the gastro-intestinal absorption of enoxacin was good, with little interpatient variability. However, no formal quantitative assessment was given, which makes it unclear on what parameters this conclusion was based. Also, no comparison was made between the extent of absorption in the febrile and non-febrile phase. The study concluded these quinolones to be an effective treatment for the investigated populations, mainly based on the sufficiently high measured serum concentrations relative to the measured MIC values of the isolated pathogens.

## Discussion

We systematically reviewed the literature on the oral bioavailability of antibiotics during the initial phase of infection in non-ICU patients to assess the possibility of an earlier IV-to-oral switch in these patients. Our review identified 9 studies on 6 antibiotics, which had in general a high risk of bias and did not provide sufficient information to compare bioavailability in febrile versus afebrile patients [21–29]. Consequently, assessments for the majority of antibiotics included in the review were uninformative. Studies on clarithromycin (*n* = 1) and ciprofloxacin (*n* = 2), where the same patients in the febrile and afebrile phase could be compared, were the only ones that provided an indication for the absence of an effect of acute illness on antibiotic bioavailability [26, 27, 29]. Although these studies had a low risk of bias, they included a very limited number of patients (*n* ≤ 12). Our review therefore indicates that insufficient evidence exists to draw a sound conclusion on whether or not the bioavailability is altered in the febrile phase relative to the afebrile phase in non-ICU patients, and as such identified a clear knowledge gap.

The six studies that compared the PK of the initial phase of infection to previous reported PK values in healthy volunteers, or that had no comparison at all, should be interpreted with caution, not only because they all had a high risk of bias, but also for the following reasons [21–25, 28].

First, when comparing PK values with previously reported PK values, as was the case for the studies on ciprofloxacin and azithromycin [21, 28], it is unclear to which extent the study population and setting are comparable. Both studies observed lower serum levels than previously reported. Bohte presented only 2 PK measurements per dosing interval, which increases the risk that the true Cmax and Tmax could not be accurately estimated [21]. This is likely to contribute to an unreliable comparison with observed values of Cmax and Tmax in healthy volunteers. In addition, the number of subjects (*n* = 8) seems not sufficient to draw a sound conclusion on the absorption of azithromycin during the initial stage of an infectious disease. The low serum concentrations of ciprofloxacin reported by Ramirez appeared not to be explained by the infectious state of the patients, since they were low on all measurement days [28].

Second, most PK studies in healthy volunteers were performed while the antimicrobial concentrations had reached steady state, rather than after a dose on the first day of treatment [33].

Third, the non-comparison studies, regarding amoxicillin, ampicillin, ciprofloxacin and enoxacin, were not designed to draw any conclusions on the bioavailability of orally administered antimicrobial agents. The primary aim of these studies was to assess their target attainment in the acutely ill phase. Yet, none of the studies defined the target to be attained. Also, the studies on ß-lactam antibiotics [22, 25] did not comment on the duration that the serum concentrations were above the MIC.

### Strengths and limitations

To the best of our knowledge, this is the first study that systematically reviewed the bioavailability of orally administered antibiotics during the initial phase of infection in non-ICU patients in accordance with the PRISMA statement. A major strength of our systematic review is that we had a very broad and thorough search strategy, and three title and abstract screening reviewers, reducing the risk that articles have been missed. Our systematic review is also subject to several limitations. Because we included studies that used very heterogeneous methods, study endpoints and outcome measurements, we were not able to pool the data and process them in a meta-analysis. Also, most of the included studies were dated from 1975 to 1995. It is uncertain whether the used laboratory methods to measure the antibiotic serum concentrations were sensitive enough to present reliable results. Most studies did not report whether their method for antibiotic concentration measurement was validated (Table 2)*.* Finally, it is possible that relevant pharmacokinetic data have not been published. For example, studies sponsored or performed by a pharmaceutical company are less likely to be published, regardless of the results [34].

### Future research

Our findings showed that knowledge of the bioavailability of orally administered antibiotics during the acute phase of a febrile illness in non-ICU patients is scarce. In previous studies the PK of antibiotics in a broader sense, so not only bioavailability, but also clearance and volume of distribution, has been mainly investigated in healthy volunteers and in critically ill patients [8–10, 35]. Non-ICU patients cannot be automatically equated with the latter, as systemic infection might profoundly alter the PK of antibiotics depending on its severity [35]. For example, in critically ill infectious patients an increased volume of distribution is often seen, due to capillary permeability negatively impacting exposure [7–10]. And in terms of clearance, as stated before, both toxic antibiotic serum concentrations and subtherapeutic antibiotic serum concentrations can be seen, depending on the perfusion alterations of the kidney. Especially in cases of augmented renal clearance both the area-under-the-concentration-time curve as well as the percentage of time of a dosing interval the antibiotic concentration is above the minimum inhibitory concentration will be lower [7–10]. In addition, the results from critically ill patients do not learn anything about bioavailability of antibiotics, as antibiotics are almost always administered intravenously in these patients. The same accounts for patients newly admitted to a general ward with an acute infection. This reluctance to administer antibiotics orally from the beginning of a course proves that clinicians are not a priori convinced that the acute phase of an infection does not alter bioavailability, neither in ICU patients nor in non-ICU patients, even if the gastrointestinal tract of the patient is intact. The two studies on ciprofloxacin [27, 29] and the one study on clarithromycin [26] in which the same patients in the febrile and afebrile phase of infection were compared, suggest that bioavailability is not altered during the initial phase of infection. Although these results are promising concerning the possibility to switch from IV to oral therapy within the first 24 h of treatment, the value of these studies is limited due to their small number of included patients (respectively *n* = 12, 7 and 12). For the other orally administered antibiotics evidence is completely lacking whether febrile illnesses influences the extent of bioavailability [21–25, 28]. If febrile illnesses do have an effect on the bioavailability of orally administered antibiotics, it is likely that there are clinical consequences of oral administration: reduced bioavailability could lead to insufficient serum concentrations, negatively affecting clinical outcome and the risk of development of antibiotic resistance, while increased bioavailability might increase the risk of toxicity [12, 36, 37]. We therefore believe that current and new oral antimicrobial agents should be tested in non-ICU patients during the acute phase of febrile illness as well, and not only in healthy volunteers or during the convalescent state.

## Conclusion

There is a clear knowledge gap regarding the bioavailability of widely used orally administered antibiotics during the initial phase of a systemic infection in non-ICU patients, as only a few, mostly small, studies could be identified on this matter that generally had a high risk of bias. Although from a theoretical perspective there does not seem to be a reason not to start early oral antibiotic therapy in febrile patients without gastrointestinal problems, this gap needs to be covered to indeed provide the evidence that an early switch (within 24 h of start of therapy) still ensures high enough antibiotic concentrations for effective treatment. Therefore, well-designed and large enough studies on this specific topic are warranted so that it can be elucidated whether patients can benefit from the advantages of the IV-to-oral switch earlier than nowadays.

## Supplementary Information

**Additional file 1.**

## Data Availability

The datasets used and/or analysed during the current study are available from the corresponding author on reasonable request.

## Abbreviations

AUC: Area-under-the-plasma-concentration-time curve
Cmax: Maximum serum concentrations
Cpeak: Peak concentrations
Cserum: Serum concentrations
Cthrough: Through concentrations
CYP: Cytochrome P450
F: Bioavailability
ICU: Intensive Care Unit
IV: Intravenous
MIC: Minimum inhibitory concentration
NOS: Newcastle-Ottawa Quality Assessment Scale
PK: Pharmacokinetics
PK/PD: Pharmacokinetics/pharmacodynamics
PPK: Population pharmacokinetics
Tmax: Time of achieving maximum serum concentrations
